# A novel stop-gain pathogenic variant in *FLT4* and a nonsynonymous pathogenic variant in *PTPN11* associated with congenital heart defects

**DOI:** 10.1186/s40001-022-00920-8

**Published:** 2022-12-10

**Authors:** Avisa Tabib, Taravat Talebi, Serwa Ghasemi, Maryam Pourirahim, Niloofar Naderi, Majid Maleki, Samira Kalayinia

**Affiliations:** 1 Heart Valve Diseases Research Center, Rajaie Cardiovascular Medical and Research Center, Iran University of Medical Sciences, Tehran, Iran; 2 Rajaie Cardiovascular Medical and Research Center, Iran University of Medical Sciences, Tehran, Iran; 3 Department of Biology, Science and Research Branch, Islamic Azad University, Tehran, Iran; 4 Cardiogenetic Research Center, Rajaie Cardiovascular Medical and Research Center, Iran University of Medical Sciences, Tehran, Iran

**Keywords:** Congenital heart defect, CHDs, Whole-exome sequencing, *FLT4*, *PTPN11*, VEGFR3

## Abstract

**Background:**

Congenital heart defects (CHDs) are the most common congenital malformations, including structural malformations in the heart and great vessels. CHD complications such as low birth weight, prematurity, pregnancy termination, mortality, and morbidity depend on the type of defect.

**Methods:**

In the present research, genetic analyses via whole-exome sequencing (WES) was performed on 3 unrelated pedigrees with CHDs. The candidate variants were confirmed, segregated by PCR-based Sanger sequencing, and evaluated by bioinformatics analysis.

**Results:**

A novel stop-gain c.C244T:p.R82X variant in the *FLT4* gene, as well as a nonsynonymous c.C1403T:p.T468M variant in the *PTPN11* gene, was reported by WES. *FLT4* encodes a receptor tyrosine kinase involved in lymphatic development and is known as vascular endothelial growth factor 3.

**Conclusions:**

We are the first to report a novel c.C244T variant in the *FLT4* gene associated with CHDs. Using WES, we also identified a nonsynonymous variant affecting protein-tyrosine phosphatase, the non-receptor type 11 (*PTPN11*) gene. The clinical implementation of WES can determine gene variants in diseases with high genetic and phenotypic heterogeneity like CHDs.

## Introduction

Congenital heart defects (CHDs) are common defects that are present at birth, accounting for 4 to 8 cases per 1000 births (about one-third of neonatal mortalities) [1, 2]. Moreover, CHDs include structural malformations in the heart and great vessels that occur during the development of the fetus [3]. These diseases have various symptoms depending on the types of defects, which are thought to have resulted from a combination of genetic and environmental factors [4]. Notably, atrioventricular septal defects, ventricular septal defects, atrial septal defects, patent ductus arteriosus, and the tetralogy of Fallot are the most common types of CHDs [5]. The tetralogy of Fallot (OMIM disease 187,500) is the most common cyanotic heart defect, accounting for approximately 5% to 10% of CHD cases [6]. With an estimated incidence of 3/10000 births, the tetralogy of Fallot presents with a combination of abnormalities such as pulmonary valve stenosis, right ventricular hypertrophy, ventricular septal defects, and the overriding aorta [6, 7]. Pulmonary valve stenosis (OMIM disease 265,500) is mostly congenital and comprises 7% to 12% of CHD cases [8, 9]. It is a relatively common defect of the pulmonic valve that is most often associated with congenital cardiac syndromes like the tetralogy of Fallot, congenital rubella, and Noonan syndrome [9]. Ventricular septal defects constitute one of the most common CHDs, and they appear with or without several complex abnormalities that can be detected between the prenatal period and adulthood [10]. Pulmonary atresia with ventricular septal defects (178,370) is a complex cyanotic CHD with various clinical symptoms based on the anatomy of the central pulmonary arteries and pulmonary distribution [11]. Aortic valve stenosis is the most common valvular cardiac defect and affects nearly 2% of the population aged over 65 years [12]. Although this progressive disease usually occurs in persons over 65 years of age, it can also occur in younger individuals with rheumatic heart disease or congenital valve abnormalities [13]. Many genes have been reported to be involved in CHD etiology [14]. Vascular endothelial growth factor 3 (VEGFR3) is encoded by the fms-like tyrosine kinase-4 (*FLT4*; 136,352) gene, involved in the development of the lymphatic system. *FLT4* variants cause Milroy disease (153,100), one of the main forms of hereditary primary lymphedema, and they also predispose to the tetralogy of Fallot, indicating the role of VEGFR3 in the primary development of the heart [15]. The protein-tyrosine phosphatase, non-receptor type 11 (*PTPN11*; 176,876) gene encodes the non-receptor type protein tyrosine phosphatase SHP-2 (src homology region 2 domain phosphatase–2), which accounts for nearly 50% of cases with Noonan syndrome. The syndrome (OMIM 163,950) is associated with a wide spectrum of CHDs such as pulmonary valve stenosis, hypertrophic cardiomyopathy, aortic coarctation, and atrioventricular septal defects [16, 17].

In this research, we utilized whole-exome sequencing [18] to determine the genetic causes of CHDs in 3 unrelated pedigrees and succeeded in identifying a novel heterozygous pathogenic stop-gain variant in exon 3 of the *FLT4* gene in an Iranian family with CHDs. Moreover, with the aid of WES, we identified a pathogenic nonsynonymous variant (c.C1403T:p.T468M) in exon 12 of the *PTPN11* gene in another Iranian family with CHDs.

## Methods

### Ethics approval and consent to participate

The present research was conducted in accordance with the Declaration of Helsinki. Ethical approval was obtained from the Ethics Committees of Rajaie Cardiovascular Medical and Research Center, Iran University of Medical Sciences, Tehran, Iran (IR.RHC.REC.1399.017). Informed consent was obtained from the probands’ parents and/or legal guardians for study participation.

### Study subjects and samples

**Fig. 1:**
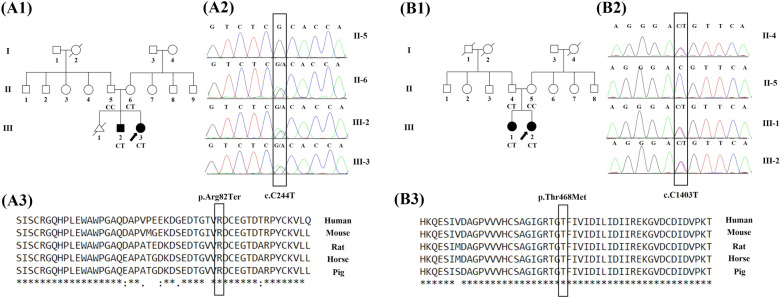
The image presents the pedigree of 2 families with congenital heart defects (CHDs), sequencing chromatograms, and the conservation analysis of the mutated amino acids in the *FLT4* and *PTPN11* genes. **A** The image depicts Family I. **A1** The image demonstrates the pedigree of Family I with individuals suffering from CHDs. The black arrow indicates the proband, and the affected and unaffected individuals are presented with filled and clean symbols, respectively. **A2** The Sanger-based sequencing results in Family I show a heterozygous variant in the *FLT4* gene in the proband (III-3) and her affected brother (III-2). The c.C244T variant in the *FLT4* gene was detected in her healthy mother (II-6), while her father (II-5) did not carry this variant. The candidate variant was validated by Sanger-based sequencing using the reverse primer. **A3** In Family I, the conservation of the p.R82X variant is shown. The variant site is highly conserved in various species. **B** The image depicts Family II. **B1** The image demonstrates the pedigree of Family II with individuals suffering from CHDs. The black arrow indicates the proband, and the affected and unaffected individuals are represented by filled and clean symbols, respectively. **B2** The Sanger-based sequencing results in Family II show a heterozygous variant in the *PTPN11* gene in the proband (III-2) and her affected sister (III-1). The c.C1403T variant in the *PTPN11* gene was detected in her healthy father (II-4), while her mother (II-5) did not carry this variant. The candidate variant was validated by Sanger-based sequencing using the forward primer. **B3** In Family II, the conservation of the p.T468M variant is shown. The variant site is highly conserved in various species

**Fig. 2:**
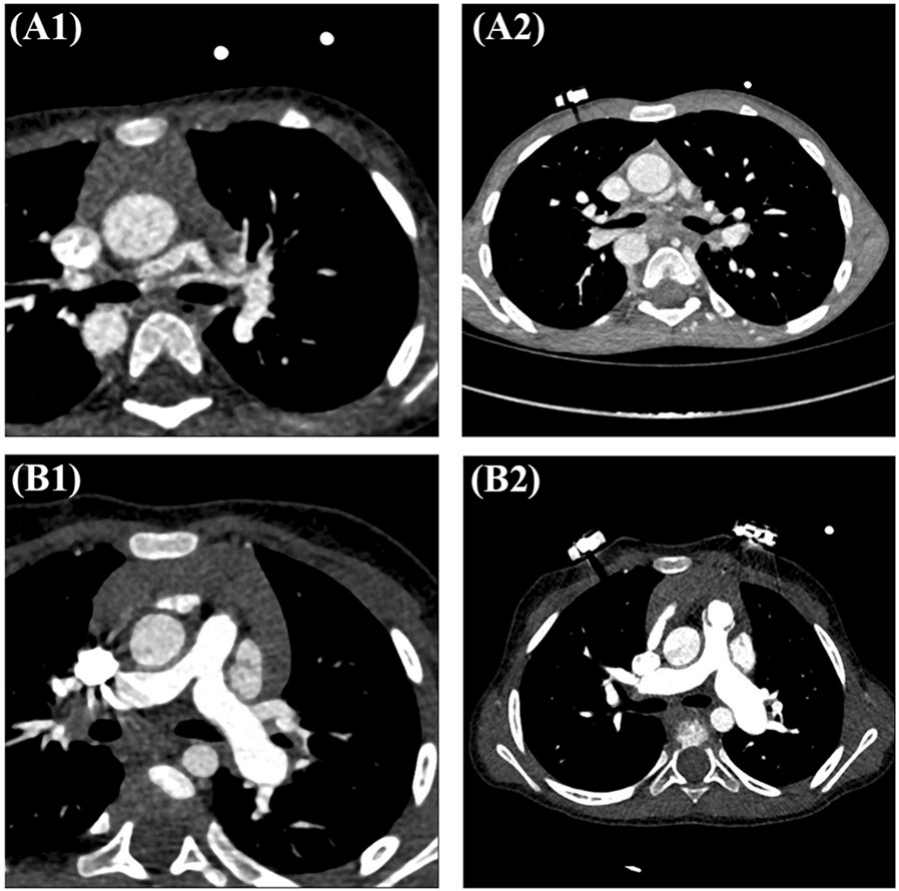
Computed tomography angiography results are presented herein. **A1** The axial view shows a dilated aorta, an atretic main pulmonary artery, and hypoplastic confluent pulmonary arterial branches. **A2** The axial view demonstrates a dilated Aorta, pulmonary atresia, and hypoplastic pulmonary arterial branches. **B1** The axial view illustrates severe PS and ASD. **B2** The axial view reveals severe pulmonary valve stenosis, a ventricular septal defect, and an atrial septal defect

Three families with CHDs were selected for genetic analysis. The genetic causes of CHDs were detected in 2 pedigrees (Fig. 1(. For further information on the cardiovascular anatomy, computed tomography angiography was performed with a Siemens SOMATOM Definition Flash 128-Slice Dual Source CT Scanner (Fig. 2). After CHD diagnosis, written informed consent was obtained from the families. Next, peripheral blood collection was performed on each study subject. Genomic DNA was then obtained from the whole-blood sample with a Cinna Pure DNA Kit (CinnaGen, Tehran, Iran). Afterward, the extracted DNA was evaluated with a NanoDrop 2000 (Thermo Fisher Scientific, Waltham, MA, USA).

### WES, validation, and search strategy

WES was performed on DNA samples from each pedigree’s proband (Fig. 1A1, B1) at Macrogen (Seoul, South Korea) with a SureSelect XT Library Prep Kit (Agilent Technologies, CA, USA) on an Illumina HiSeq 4000 (Illumina, San Diego, USA). With the aid of the BWA software package, qualified reads were aligned to the hg19 version of the human reference genome. Subsequently, the Genome Analysis Toolkit (GATK) was applied to access variant calling. Functional annotation of the detected variants was performed using the ANNOVAR software tool. The effects of the variants on protein functions were predicted using MutationTaster, MutationAssessor, PolyPhen, SIFT, and CADD. In the next stage, variants with a minor allele frequency below 5% in the 1000 Genomes Project, ESP6500, and ExAC databases were filtered out. Next, all the candidate variants were validated by targeted polymerase chain reaction (PCR) amplification and Sanger sequencing on available family members (Applied Biosystems 3500G, Foster City, CA, USA). The specific primers were designed with the Geneious software (Geneious 10.2.2, Biomatters Ltd).

In the first family study, 2 specific primers were constructed: 1 forward primer (5′-GGTCATCTGCATCCACTCC-3′) and 1 reverse primer (5′-ATGCGTGCCTTGATGTACTTGT-3′). In the second family study, 2 specific primers were constructed: 1 forward primer (5ʹ-ACTCTGTATGGTATGTGCTGTTG-3′) and 1 reverse primer (5′-GAAGTGGCAGAAGTCAGGATT-3′). A literature search was conducted by searching Google Scholar, ClinVar, HGMD, and PubMed to gather the detected variants in the identified genes related to CHDs. The search was performed utilizing the keywords that included the clinical significance of *FLT4* variants, the clinical significance of *PTPN11* variants, *FLT4* variants, and *PTPN11* variants.

### Homology modeling and docking

#### FLT4

**Fig. 3:**
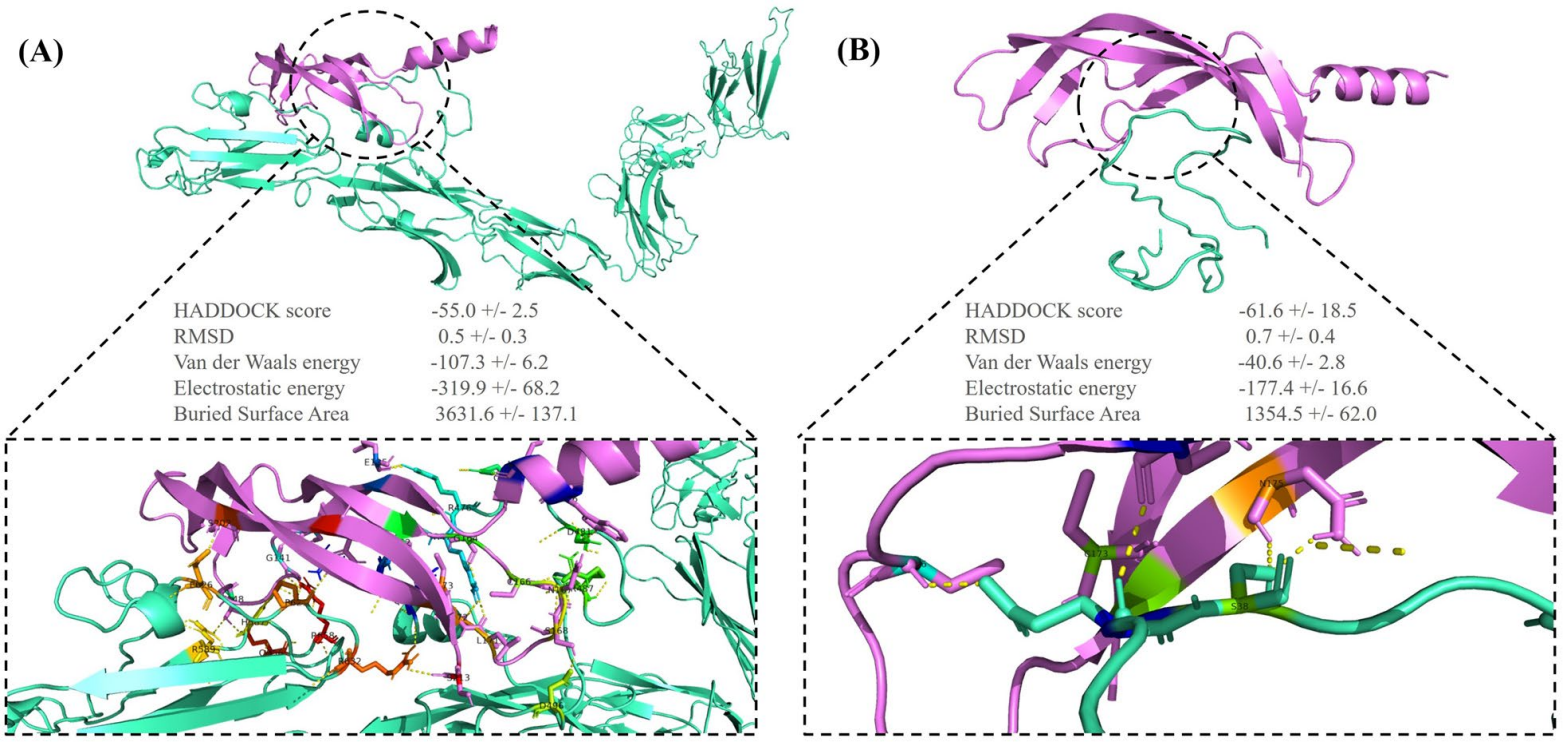
The image illustrates the binding pattern of the normal/mutant vascular endothelial growth factor 3 (VEGFR3) with vascular endothelial growth factor C (VEGFC). **A** The image presents the 3D interaction between the normal VEGFR3 (green) and VEGFC (violet). **B** The image illustrates the 3D interaction between the mutant VEGFR3 (green) and VEGFC (violet). These visualizations were obtained using the PyMOL software

**Fig. 4:**
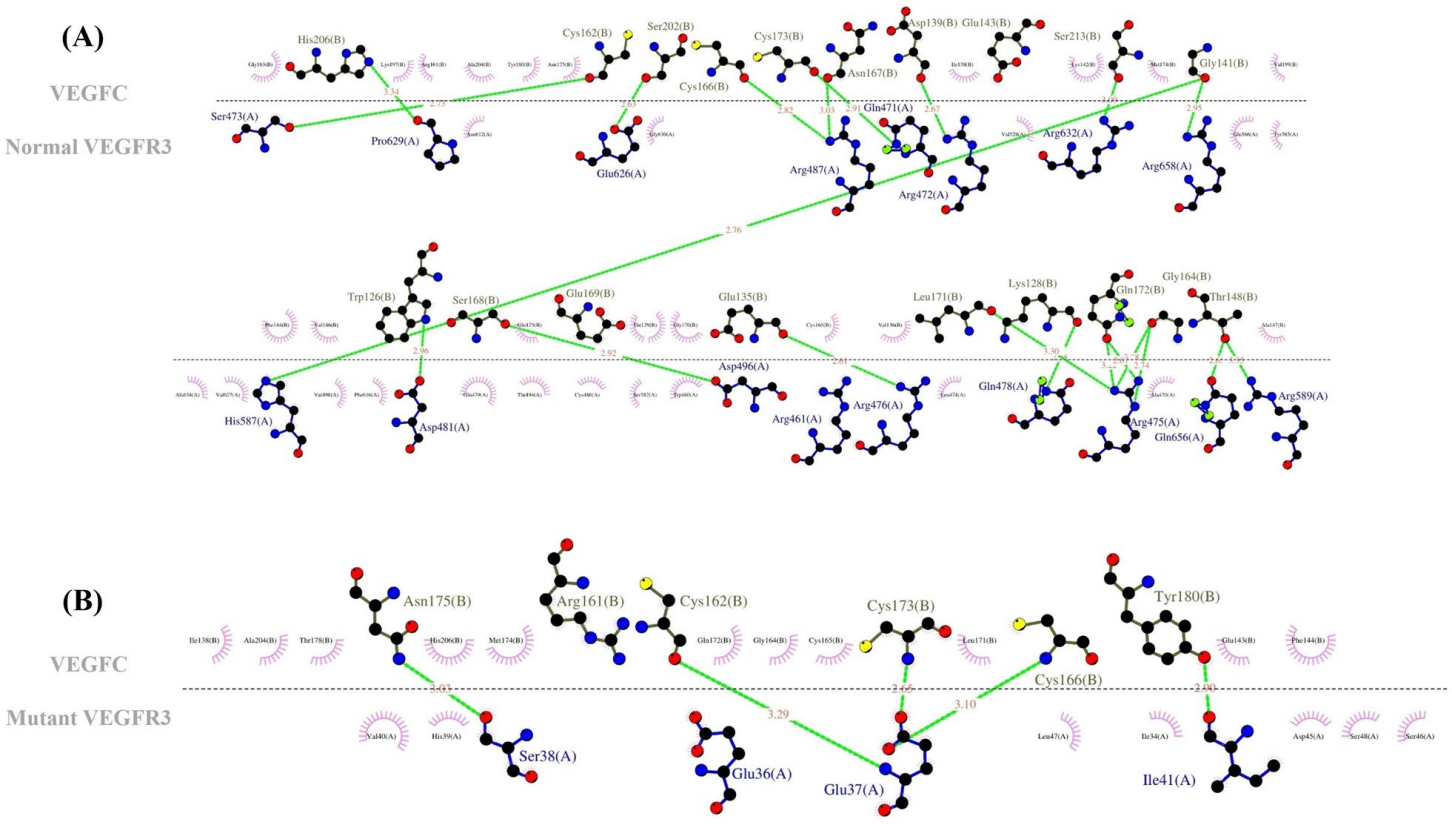
The image presents a 2D image of ligand–protein interactions. **A** The image illustrates the interaction between the normal VEGFR3 and VEGFC. **B** The image depicts the interaction between the mutant VEGFR3 and VEGFC. These images were generated by LigPlus + . (The green dashed lines represent hydrogen bonds with bond distance.)

*FLT4* encodes the VEGFR3 protein, which plays a significant role in embryonic blood vascular development and postnatal angiogenesis. Defects in any members of VEGF signaling, as a novel and plausible pathomechanism of the tetralogy of Fallot, are related to cardiovascular defects. The stop-gain variant in *VEGFA* and loss of function in *FLT4* is a candidate for the tetralogy of Fallot pathogenesis. VEGFR3 homodimers respond to signaling induced by vascular endothelial growth factor C (VEGFC) and regulate the development of the lymphatic vasculature. In return, VEGFR3/VEGFR2 heterodimers are induced by VEGFA and VEGFC and are involved in the regulation of sprouting angiogenesis and vascular network formation [19]. According to a previous study, in the absence of VEGFR3 in knockout murine samples, a higher abundance of VEGFC might cause signaling only through VEGFR2, leading to the disruption of blood cell formation and angiogenesis during embryogenesis [20]. The effects of normal/mutant VEGFR3 on VEGF signaling are illustrated in Figs. 3 and 4.

#### PTPN11

**Fig. 5:**
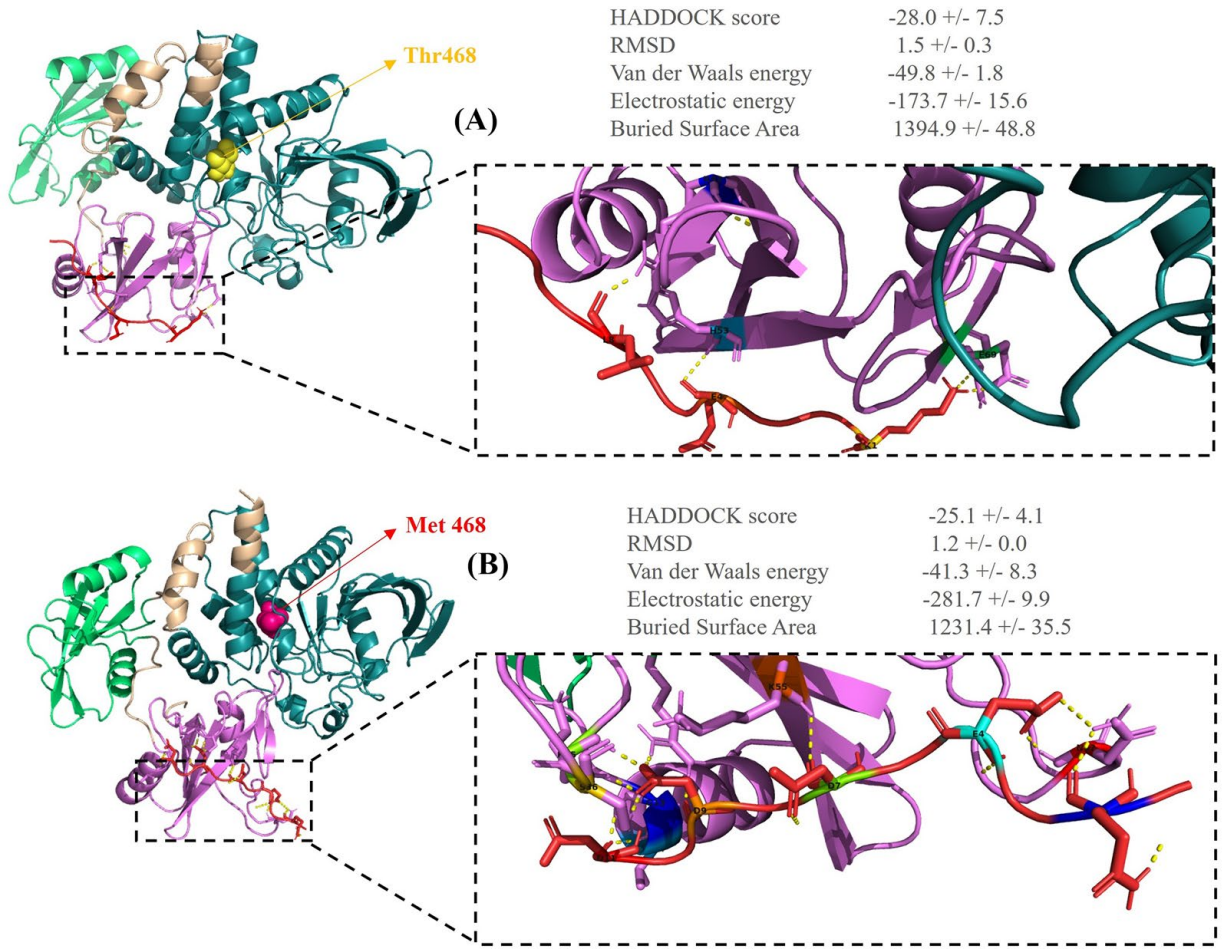
The binding pattern of the normal/mutant src homology region 2 domain phosphatase-2 (SHP-2) with Grb2-associated binder-1 (Gab-1) is presented herein. **A** The image shows the 3D interaction between the normal SHP-2 (N-SH2 domain: violet, C-SH2 domain: limo green, PTP domain: teal) and Gab-1 (red). **B** The image demonstrates the 3D interaction between the mutant SHP-2 (N-SH2 domain: violet, C-SH2 domain: limo green, PTP domain: teal) and Gab-1 (red). These visualizations were obtained using the PyMOL software

**Fig. 6:**
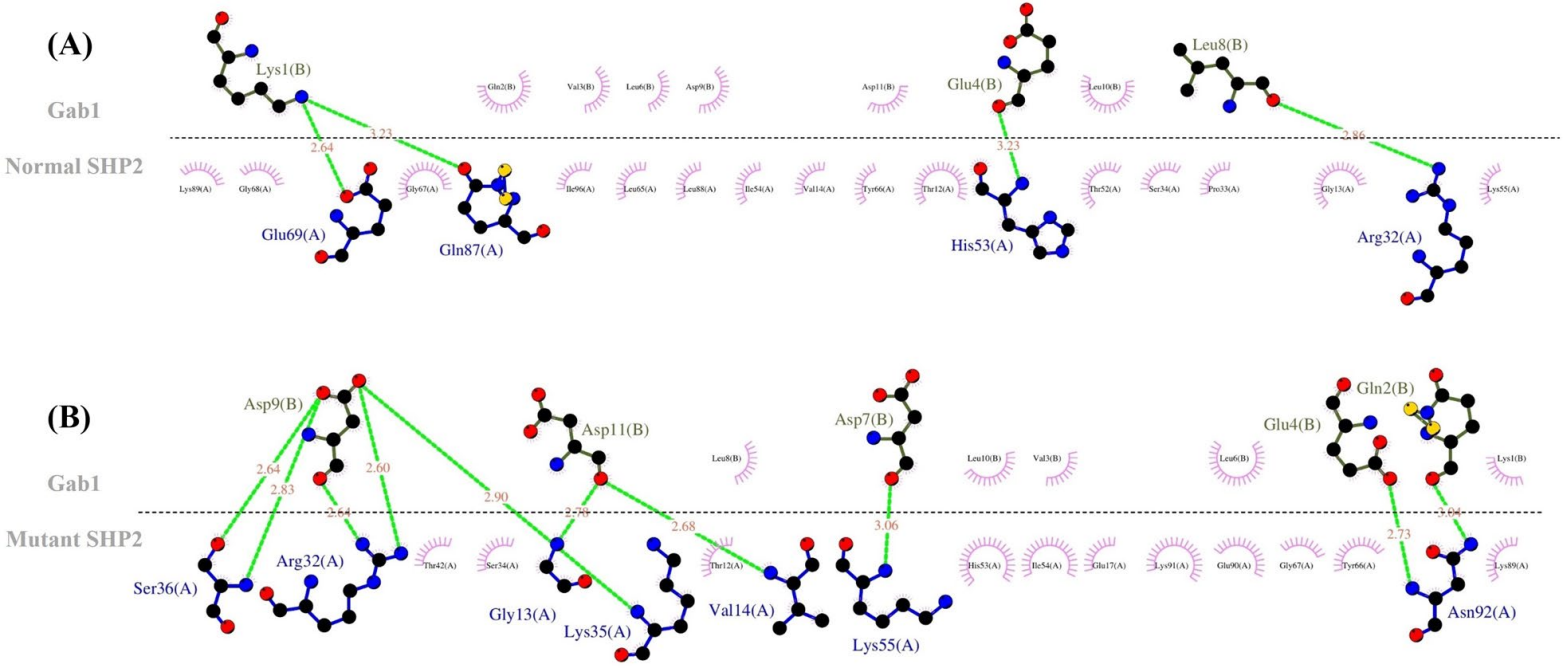
The 2D image of ligand–protein interactions is illustrated herein. **A** The image demonstrates the interaction between the normal SHP-2 and Gab-1. **B** The image shows the interaction between the mutant SHP-2 and Gab-1. These images were generated by LigPlus + . (The green dashed lines represent hydrogen bonds with bond distance.)

*PTPN11* encodes the SHP-2 protein, which, as a phosphatase, has a critical role in the activation of the RAS mitogen-activated protein kinase (MAPK) pathway by phosphorylation mechanism. The RAS–MAPK signaling pathway is involved in embryonic development during weeks 2 to 8, the critical cardiac development period. Hence, any disturbance to this pathway is likely to cause heart defects [21]. The SHP-2 protein has an auto-inhibited state in the closed structure by N-SH2–PTP inter-domain interaction. The substitution of Met for Thr468 in the PTP domain weakens intramolecular N-SH2–PTP interactions, destabilizes the SHP-2 closed structure, and increases the SHP-2 open form, enhancing the binding of N-SH2 with phosphorylated tyrosine in partners. The major SHP-2 binding partner is Grb2-associated binder-1 (Gab-1), which is bound by SHP-2 to its phosphorylated tyrosine through the N-SH2 domain. T468M SHP-2 lengthens the binding of SHP-2 to Grb1, which probably leads to the further phosphorylation and activation of the RAS–ERK1/2 pathway [22, 23]. The effects of normal/mutant SHP-2 on the RAS–MAPK signaling pathway are depicted in Figs. 5 and 6.

#### Modeling and docking methods

In the present study, the Protein Data Bank (PDB) (https://www.rcsb.org/) was drawn upon to obtain the crystal structure of SHP-2 (normal and mutant), Gab-1, and VEGFC. Additionally, through the use of the homology-modeling server of SWISS-MODEL (https://swissmodel.expasy.org/), 3D structures of VEGFR3 (normal and mutant) were created. The protein structures were corrected by ViewerLite (v.1.5.1). In other words, all heteroatoms, consisting of water molecules, ions, and native ligands, were deleted, and polar hydrogens were added. Then, the energy of the models was minimized using the YASARA minimization server (http://www.yasara.org/). The SCE files obtained from the YASARA minimization server were imported into YASARA View (v.20.12.24) to be saved as PDB files. For the prediction of the binding site, the server of the Computed Atlas of Surface Topography of proteins (CASTp) (http://sts.bioe.uic.edu/) was employed. Docking was also performed by using the HADDOCK web server (https://wenmr.science.uu.nl/haddock2.4/). Thereafter, the interactions among the conformations were analyzed using PyMOL (v.2.5.2) and LigPlus + (v.2.2.4). After the completion of docking, the lowest HADDOCK score and the root mean square deviation conformation were considered the most suitable docking pose for the compounds.

## Results

### Clinical analysis

The computed tomography angiography findings indicated that both patients of Family I (Fig. 2A1, A2) had severe cyanosis in the neonatal period. Echocardiography revealed pulmonary atresia and a large ventricular septal defect in both cases. Multiple coronal, axial, and sagittal images revealed a large subaortic ventricular septal defect and pulmonary atresia with hypoplastic pulmonary artery branches and multiple aortopulmonary collaterals in both patients. The patients of Family II (Fig. 2 B1, B2) had a heart murmur in infancy. Echocardiography revealed pulmonary stenosis, a large ventricular septal defect, and an atrial septal defect in the first case and pulmonary valve stenosis and an atrial septal defect in the second case.

### Molecular analysis

The current study enrolled 3 distinct pedigrees with various patterns of inheritance. The novel pathogenic c.C244T:p.R82X variant in the *FLT4* gene was detected in the first pedigree, and it segregated with CHDs in this family. In the second pedigree, the heterozygous c.C1403T:p.T468M variant in the *PTPN11* gene was identified. The 2 pedigrees are presented in Fig. 1. The proband of the third pedigree was a 3-year-old girl with heart murmurs and cyanosis. The initial analysis showed that the identified variants in this pedigree were variants of uncertain significance. These variants in 5 genes (*AXIN2*, *EGF*, *PFKL*, *PKHD1L1*, and *PIEZO1*) did not segregate with the examined defect in this pedigree.

The reported pathogenic variants in the *FLT4* gene related to lymphedema and Milroy disease are summarized in Table 1 [19, 24–39], and the reported pathogenic variants in the *PTPN11* gene related to Noonan syndrome and LEOPARD syndrome are summarized in Table 2 [40–55]. The functional significance of the identified genetic variants was assessed through bioinformatics analysis using CADD, SIFT, PROVEAN, MutationTaster, and PolyPhen-2. According to the collected data, the p.Q736X variant in the *FLT4* gene had the highest CADD number in Table 1. Likewise, the p.D61N, p.E110K, p.F285L, p.F285C, p.F285S, p.A461S, p.A461T, p.S502L and P.F285I variants in the *PTPN11* gene had the highest CADD number in Table 2.

**Table 1:** Bioinformatics analysis of pathogenic reported variants in *FLT4* (NM_182925.5) related to congenital heart defects

No.	Exon	HGVS DNA	HGVS Protein	Chromosome 5 Position (hg19)	dbSNP	CADD	SIFT	Polyphen-2	PROVEAN	Mutation taster	ClinVar	Condition	Refs.
1	18	c.2554G > A	p. G852S	180,046,758	NA	26.8	D	PD	D	DC	NA	MD	[24]
2	18	c.2554G > C	p. G852R	180,046,758	NA	26.7	D	PD	D	DC	NA	MD	[24]
3	18	c.2555G > T	p. G852V	180,046,757	NA	26.1	D	PD	D	DC	NA	MD	[24]
4	18	c.2560G > C	p. G854R	180,046,752	rs144045237	29.2	D	PD	D	DC	NA	MD	[24,25]
5	18	c.2569G > A	p. G857R	180,046,743	rs267606818	27.9	D	PD	D	DC	P	MD	[24,26]
6	26	c.3433C > T	p.R1145C	180,039,610	rs202140363	25.9	D	PD	N	DC	NA	MD	[24]
7	19	c.2740G > C	p. G914R	180,046,274	NA	32	D	PD	D	DC	NA	MD	[24]
8	19	c.2744C > A	p. A915E	180,046,270	NA	32	D	PD	D	DC	NA	MD	[24]
9	19	c.2749A > C	p. T917P	180,046,265	NA	29.4	D	PD	D	DC	NA	MD	[24]
10	20	c.2797G > C	p. G933R	180,046,074	NA	32	D	PD	D	DC	NA	MD	[24,25]
11	20	c.2800A > T	p. N934Y	180,046,071	NA	32	D	PD	D	DC	NA	MD	[24]
12	20	c.2828G > C	p. R943P	180,046,043	NA	31	D	PD	D	DC	NA	MD	[24]
13	22	c.3054C > A	p. S1018R	180,043,942	NA	25.4	D	PD	D	DC	NA	MD	[24]
14	22	c.3056 T > C	p. F1019S	180,043,940	NA	30	D	PD	D	DC	UVS	MD	[24]
15	22	c.3075G > A	p. M1025I	180,043,921	NA	29.8	D	PD	D	DC	NA	MD	[24]
16	23	c.3097 T > G	p. C1033G	180,043,489	NA	27.9	D	PD	D	DC	NA	MD	[24]
17	23	c.3104A > G	p. H1035R	180,043,482	rs121909653	25.4	D	PD	D	DC	P	MD	[24]
18	23	c.3109G > C	p. D1037H	180,043,477	NA	28.9	D	PD	D	DC	NA	MD	[24]
19	23	c.3111C > A	p. D1037E	180,043,475	NA	24.5	D	PD	D	DC	NA	MD	[24]
20	23	c.3119C > A	p. A1040D	180,043,467	NA	27.6	D	PD	D	DC	NA	MD	[24]
21	23	c.3122G > A	p. R1041Q	180,043,464	NA	29.8	D	PD	D	DC	NA	MD	[24]
22	23	c.3122G > C	p. R1041P	180,043,464	rs121909650	28.9	D	PD	D	DC	P	MD	[24,26,27]
23	23	c.3157A > T	p. I1053F	180,043,429	NA	27.1	D	PD	D	DC	NA	MD	[24]
24	23	c.3170G > A	p. G1057D	180,043,416	NA	26.7	D	PD	D	DC	NA	MD	[24]
25	24	c.3250G > A	p. E1084K	180,041,149	NA	31	D	PD	D	DC	NA	MD	[24]
26	24	c.3308 T > C	p. L1103P	180,041,091	NA	26.1	D	PD	D	DC	NA	MD	[24]
27	24	c.3310C > T	p. L1104F	180,041,089	NA	25.3	D	PD	D	DC	NA	MD	[24]
28	24	c.3318G > T	p. E1106D	180,041,081	rs201431522	25.3	D	PD	D	DC	NA	MD	[24]
29	24	c.3323 T > C	p. F1108S	180,041,076	NA	29.1	D	PD	D	DC	NA	MD	[24]
30	25	c.3391G > C	p. G1131R	180,040,051	NA	27.5	D	PD	D	DC	P	MD	[24]
31	17	c.2531G > C	p. R844P	180,047,184	NA	29.5	D	PD	D	DC	NA	MD	[24]
32	18	c.2560G > A	p. G854S	180,046,752	NA	28.5	D	PD	D	DC	NA	MD	[24]
33	18	c.2563G > A	p. A855T	180,046,749	rs121909657	24.6	D	PD	D	DC	P	MD	[24,27,74]
34	18	c.2629G > A	p. A877T	180,046,683	NA	25.9	D	PD	D	DC	NA	MD	[24]
35	18	c.2632G > A	p. V878M	180,046,680	rs121909654	25.6	D	PD	D	DC	P	MD	[24,27,29]
36	19	c.2677C > G	p. L893V	180,046,337	NA	25.0	D	PD	D	DC	NA	MD	[24,30,31]
37	19	c.2737_2739del	p.Leu913del	180,046,275	NA	22.5	NA	NA	NA	DC	NA	MD	[24]
38	19	c.2743G > C	p.A915P	180,046,271	NA	32	D	PD	D	DC	NA	MD	[24,25]
39	19	c.2748C > G	p.C916W	180,046,266	NA	27.3	D	PD	D	DC	NA	MD	[24,25]
40	22	c.3059A > T	p.Q1020L	180,043,937	NA	27.4	D	PD	D	DC	NA	MD	[24,32]
41	22	c.3070G > A	p.G1024R	180,043,926	NA	29.6	D	PD	D	DC	NA	MD	[24]
42	22	c.3071G > A	p.G1024E	180,043,925	NA	28.2	D	PD	D	DC	NA	MD	[24]
43	23	c.3105C > G	p.H1035Q	180,043,481	NA	25.1	D	PD	D	DC	NA	MD	[24,29,30]
44	23	c.3109G > T	p.D1037Y	180,043,477	NA	31	D	PD	D	DC	NA	MD	[24]
45	23	c.3121C > T	p.R1041W	180,043,465	rs1451816005	27.9	D	PD	D	DC	LP	MD	[24,25]
46	23	c.3125A > G	p.N1042S	180,043,461	NA	25.0	D	PD	D	DC	NA	MD	[24]
47	23	c.3131 T > C	p.L1044P	180,043,455	rs121909651	27.2	D	PD	D	DC	P	MD	[24,26]
48	23	c.3151G > A	p.V1051M	180,043,435	NA	25.1	D	PD	D	DC	NA	MD	[24]
49	23	c.3164A > C	p.D1055A	180,043,422	NA	26.9	D	PD	D	DC	NA	MD	[24]
50	23	c.3164A > T	p.D1055V	180,043,422	NA	27.7	D	PD	D	DC	NA	MD	[24]
51	24	c.3257 T > C	p.I1086T	180,041,142	rs121909655	26.4	D	PD	D	DC	P	MD	[24,29]
52	24	c.3316G > A	p.E1106K	180,041,083	rs121909656	27.2	D	PD	D	DC	P	MD	[24,33]
53	25	c.3341C > T	p.P1114L	180,040,101	rs121909652	29.8	D	PD	D	DC	P	MD	[24,26,27,30]
54	25	c.3344A > G	p.Y1115C	180,040,098	NA	28.3	D	PD	D	DC	NA	MD	[24]
55	25	c.3410C > T	p.P1137L	180,040,032	rs1762335528	27.6	D	PD	D	DC	LP	MD	[24,25,30,31]
56	3	c.244C > T	p.R82X	180,057,711	NA	34	NA	NA	NA	DC	P	TOF	[24]
57	8	c.1083C > A	p.Y361X	180,055,902	NA	36	NA	NA	NA	DC	NA	TOF	[24]
58	15	c.2206C > T	p.Q736X	180,047,969	NA	42	NA	NA	NA	DC	NA	TOF	[24]
59	21	c.2995C > T	p.Q999X	180,045,776	NA	35	NA	NA	NA	DC	NA	TOF	[24]
60	21	c.2860C > T	p.P954S	180,045,911	rs34255532	21.3	D	B	N	DC	NA	MD	[24]
61	18	c.2587 T > C	p.S863P	180,046,725	NA	25.6	T	PD	D	DC	NA	Lymphedema	[24]
62	20	c.2819G > C	p.R940P	180,046,052	NA	32	D	PD	D	DC	NA	Lymphedema	[30]
63	25	c.3391G > A	p.G1131S	180,040,051	rs1554109707	27.7	D	PD	D	DC	LP	Lymphedema	[30]
64	13	c.1921C > T	p.P641S	180,048,641	rs55667289	22.1	T	B	N	DC	NA	PCL	[26]
65	20	c.2777 T > C	p.I926T	180,046,094	NA	32	D	PD	D	DC	NA	PCL	[34]
66	19	c.2740G > T	p.G914W	180,046,274	NA	32	D	PD	D	DC	NA	PCL	[34]
67	18	c.2575G > A	p.V859M	180,046,737	NA	26.2	D	PD	D	DC	NA	PCL	[34]
68	19	c.2650G > A	p.G884S	180,046,364	NA	29.9	D	PD	D	DC	NA	MD	[35]
69	28	c.3704C > G	p.S1235C	180,037,008	NA	29.9	T	PD	N	DC	NA	MD	[36]
70	23	c.3163G > C	p.D1055H	180,043,423	NA	27.7	D	PD	D	DC	NA	MD	[37]
71	24	c.3315G > C	p.W1105C	180,041,084	NA	28.7	D	PD	D	DC	NA	MD	[37]
72	24	c.3295 T > C	p.S1099P	180,041,104	NA	27.2	D	PD	D	DC	NA	MD	[37]
73	18	c.2515G > C	p.E839Q	180,047,200	NA	27.5	D	PD	D	DC	NA	MD	[37]
74	10	c.1289C > T	p.S430F	180,053,001	NA	23.6	T	PD	D	DC	NA	MD	[37]
75	20	c.2771 T > C	p.M924T	180,046,100	NA	29.5	D	PD	D	DC	NA	MD	[37]
76	24	c.3296C > T	p.S1099F	180,041,103	NA	29.2	D	PD	D	DC	NA	MD	[37]
77	24	c.3230C > T	p.P1077L	180,041,169	NA	28.6	D	PD	D	DC	NA	MD	[37]
78	18	c.2615G > A	p.S872N	180,046,697	NA	22.6	D	PD	N	DC	NA	MD	[37]
79	24	c.3233 T > A	p.L1078Q	180,041,166	NA	28.0	D	PD	D	DC	NA	MD	[37]
80	23	c.3175G > C	p.A1059P	180,043,411	NA	26.8	D	PD	D	DC	P	MD	[37]
81	24	c.3316G > C	p.E1106Q	180,041,083	NA	26.2	D	PD	D	DC	NA	MD	[37]
82	18	c.2546G > A	p.R849K	180,046,766	NA	14.84	T	B	N	DC	NA	MD	[37]
83	18	c.2554G > T	p.G852C	180,046,758	NA	27.8	D	PD	D	DC	NA	MD	[37]
84	2	c.137G > A	p.S46N	180,058,700	NA	22.3	D	B	N	DC	NA	MD	[37]
85	22	c.3073A > T	p.M1025L	180,043,923	NA	26.5	D	B	D	DC	NA	MD	[37]
86	23	c.3111 C > G	p.D1037E	180,043,475	NA	24.5	D	PD	D	DC	NA	MD	[38]
87	24	c.3323_3325delTCT	p.F1108del	180,041,074	rs587776833	21.1	NA	NA	NA	DC	P	MD	[24,25,29]
88	17	c.2542 + 2delT	NA	180,047,171	NA	24.3	NA	NA	NA		NA	MD	[24,35]
89	24	c.3243G > T	M1081I	180,041,156	NA	26.9	D	PD	D	DC	NA	MD	[39]
90	9	c.1258 + 6_1258 + 10 delTCAGG	NA	180,053,101	rs1247895470	3.209	NA	NA	NA	DC	NA	MD	[37]
91	12	c.1622dupG	p. Q542PTer3	180,049,766	rs1581655293	33	NA	NA	NA	DC	NA	TOF	[37]
92	2	c.89delC	p. P30R Ter3	180,058,748	rs755445139	29.1	NA	NA	NA	DC	NA	TOF	[37]

*CHD* congenital heart disease, *MD* Milroy disease, *TOF* tetralogy of Fallot, *PCL* primary congenital lymphedema, DC disease causing, *D* deleterious, *T* tolerated, *N* neutral, *B* benign, *PD* probably damaging, *P* pathogenic, *NA* not available/not applicable

CADD, Phred ≤ 20: damaging; Phred > 20: natural

SIFT, score ≤ 0.05: deleterious; score > 0.05: tolerable

Polyphen-2, score = 0–0.15: benign; score = 0.15–0.85: possibly damaging; score = 0.85–1: probably damaging

PROVEAN, score ≤ − 2.5: deleterious; score > − 2.5: natural

**Table 2:** Bioinformatics analysis of pathogenic reported variants in *PTPN11* (NM_002834) related to congenital heart defects

No.	Exon	HGVS DNA	HGVS protein	Chromosome 12 position (hg19)	dbSNP	CADD	SIFT	Polyphen-2	PROVEAN	MutationTaster	ClinVar	Condition (CHD or cardiomyopathy)	Refs.
1	1	c.5C > T	p.T2I	112,856,920	rs267606990	23.8	T	B	N	DC	P	NS (PS)	[40]
2	2	c.124A > G	p.T42A	112,884,189	rs397507501	22.7	T	PD	D	DC	P	NS (PS, ASD)	[40,41]
3	3	c.184 T > G	p.Y62D	112,888,168	rs121918460	24.9	D	PD	D	DC	P	NS (AVCD, ASD, PS), PST, PDA	[40–44]
4	3	c.188A > G	p.Y63C	112,888,172	rs121918459	28.1	D	PD	D	DC	P	NS (AVCD, HCM, PS)	[40–42]
5	3	c.215C > G	p.A72G	112,888,199	rs121918454	23.7	D	PD	D	DC	P	NS (HCM, PS)	[40,41]
6	3	c.A236C	p.Q79P	112,888,220	NA	24.0	D	PD	D	DC	NA	NS (PS)	[40,43]
7	3	c.317A > C	p.D106A	112,888,301	rs397507517	25.3	D	PD	D	DC	P	NS (ASD, PS)	[40,41,43,45]
8	7	c.836A > G	p.Y279C	112,910,827	rs121918456	28.9	D	PD	D	DC	P	LS (PVS, HCM), NS (PS,HCM)	[40–42,46–48]
9	8	c.922A > G	p.N308D	112,915,523	rs28933386	23.6	D	PD	D	DC	P	NS (PS, HCM,VSD)	[40–42,49,50]
10	8	c.923A > G	p.N308S	112,915,524	rs121918455	22.7	T	PD	D	DC	P	PS, NS (HCM)	[40,41,51]
11	12	c.1403C > T	p.T468M	112,926,270	rs121918457	28.7	D	PD	D	DC	P	LS (HCM, AVCD), NS (PS, HCM)	[40,41,46–48]
12	13	c.1508G > C	p.G503A	112,926,888	rs397507546	27.4	T	PD	D	DC	P	NS (PS)	[40]
13	13	c.1510A > G	p.M504V	112,926,890	rs397507547	26.8	T	PD	D	DC	P	NS (PS,HCM)	[[40–43]
14	14	c.1678C > T	p.L560F	112,940,026	rs397516797	22.7	D	B	N	DC	LB	NS (HCM)	[40]
15	5	c.540C > T	p.D180 =	112,892,382	rs753269427	9.256	T	NA	N	DC	LB	CHD	[52]
16	2	c.127C > T	p.L43F	112,884,192	NA	27.8	D	PD	D	DC	NA	CHD	[52]
17	3	c.155C > T	p.T52I	112,888,139	rs397507503	28.0	D	PD	D	DC	P	NS (PS)	[41]
18	3	c.172A > G	p.N58D	112,888,156	rs397507505	26.6	T	PD	D	DC	P	NS (PS)	[41,51]
19	3	c.172A > C	p.N58H	112,888,156	rs397507505	25.7	T	PD	D	DC	P	NS (PS)	[41]
20	3	c.174C > A	P.N58K	112,888,158	rs397507506	25.0	T	PD	D	DC	P	NS (PS)	[41]
21	3	c.179G > C	p.G60A	112,888,163	rs397507509	26.5	D	PD	D	DC	P	NS (PS, ASD)	[41,43]
22	3	c.178G > A	p.G60S	112,888,162	rs397507507	29.7	D	PD	D	DC	P	NS (PS)	[41]
23	3	c.182A > G	p.D61G	112,888,166	rs121918461	28.2	D	PD	D	DC	P	NS (PS,ASD)	[41,43]
24	3	c.181G > A	p.D61N	112,888,165	rs397507510	32	D	PD	D	DC	P	ASD, NS (PS)	[41–43,51]
25	3	c.214G > T	p.A72S	112,888,198	rs121918453	25.0	D	PD	D	DC	P	NS (PS,HCM)	[41,51]
26	3	c.218C > T	p.T73I	112,888,202	rs121918462	27.4	D	PD	D	DC	DC	NS (PS,ASD)	[41,43]
27	3	c.217_218delinsCT	p.T73L	112,888,201	rs397516802	28.0	D	PD	D	DC	P	NS (PS)	[41]
28	3	c.228G > C	p.E76D	112,888,212	rs397507514	22.3	D	PD	D	DC	P	NS	[41,51]
29	3	c.236A > G	p.Q79R	112,888,220	rs121918466	26.0	D	PD	D	DC	P	NS (PS)	[41–43]
30	4	c.328G > A	p.E110K	112,888,312	rs397507518	32	D	PD	D	DC	P	NS	[41]
31	4	c.417G > C	p.E139D	112,891,083	rs397507520	25.0	D	PD	D	DC	P	NS (PS), HIS	[41,53]
32	7	c.767A > G	p.Q256R	112,910,758	rs397507523	24.3	T	B	D	DC	P	NS (PS)	[41]
33	7	c.781C > T	p.L261F	112,910,772	rs397507525	22.6	T	B	N	DC	P	NS	[41]
34	7	c.802G > T	p.G268C	112,910,793	rs397507527	31	D	PD	D	DC	P	NS	[41]
35	7	c.844A > G	p.I282V	112,910,835	rs397507529	23.4	T	B	N	DC	P	NS (PS)	[41]
36	7	c.853 T > C	p.F285L	112,910,844	rs397507531	32	D	PD	D	DC	P	NS (PS)	[41]
37	8	c.854 T > G	p.F285C	112,915,455	rs121918463	32	D	PD	D	DC	P	NS (PS)	[41]
38	8	c.854 T > C	p.F285S	112,915,455	rs121918463	32	D	PD	D	DC	P	NS (PS),VSD, ASD	[41,51,53]
39	12	c.1381G > T	p.A461S	112,926,248	rs121918468	32	D	PD	D	DC	P	NS (HCM)	[41]
40	12	c.1381G > A	p.A461T	112,926,248	rs121918468	32	D	PD	D	DC	P	NS (HCM)	[41]
41	13	c.1471C > T	p.P491S	112,926,851	rs397507539	23.1	T	B	N	DC	P	NS (PS)	[41]
42	13	c.1471C > A	p.P491T	112,926,851	rs397507539	23.1	T	PD	N	DC	P	NS (PS)	[41]
43	13	c.1504 T > G	p.S502A	112,926,884	rs121918458	26.9	T	PD	D	DC	P	NS (PS)	[41]
44	13	c.1505C > T	p.S502L	112,926,885	rs397507544	32	T	PD	D	DC	P	NS (PS)	[41]
45	13	c.1528C > G	p.Q510E	112,926,908	rs397507549	26.5	D	PD	D	DC	P	NS (PS, HCM),VSD	[41,48,54]
46	13	c.1529A > C	p.Q510P	112,926,909	rs121918470	27.9	D	PD	D	DC	P	NS (PS)	[41]
47	13	c.1529A > G	p.Q510R	112,926,909	rs121918470	27.5	D	PD	D	DC	P	NS (PS, HCM)	[41,42]
48	3	c.166A > G	p.I56V*	112,888,150	rs397507504	23.6	D	PD	N	DC	P	HCM	[42]
49	3	c.205G > C	p.E69Q	112,888,189	rs397507511	25.5	D	PD	D	DC	P	NS	[42,45]
50	7	c.846C > G	p.I282M*	112,910,837	rs397507530	23.2	D	PD	D	DC	P	NS	[42]
51	3	c.181G > C	p.D61H	112,888,165	rs397507510	29.2	D	PD	D	DC	P	NS (AVSD)	[53]
52	13	c.1472C > T	p.P491L	112,926,852	rs397507540	23.7	T	PD	D	DC	P	NS	[45]
53	3	c.317A > G	p.D106G	112,888,301	NA	29.3	D	PD	D	DC	P	NS	[45]
54	13	c.1492C > T	P.R498W	112,926,872	rs397507541	31	D	PD	D	DC	P	LS (HCM)	[46]
55	13	c.1493G > T	P.R498L	112,926,873	rs397507542	31	D	PD	D	DC	P	LS (HCM)	[46]
56	13	c.1517A > C	P.Q506P	112,926,897	rs397507548	27.2	D	PD	D	DC	P	LS (PS)	[46]
57	7	c.836A > C	P.Y279S	112,910,827	rs121918456	27.7	D	PD	D	DC	P	LS (HCM)	[46,47]
58	7	c.853 T > A	p.F285I	112,910,844	rs397507531	32	D	PD	D	DC	P	NS	[51]
59	12	c.1391G > C	p.G464A	112,926,258	rs121918469	28.4	D	PD	D	DC	P	NS	[51]
60	13	c.1507G > A	p.G503R	112,926,887	rs397507545	31	T	PD	D	DC	P	NS (PS)	[41,51]
61	13	c.1508G > A	p. G503E	112,926,888	rs397507546	29.3	T	PD	D	DC	P	NS (PS)	[51]
62	13	c.1510A > T	p.M504L	112,926,890	NA	23.3	T	B	N	DC	NA	NS	[51]
63	13	c.1471C > G	p.P491A	112,926,851	rs397507539	22.6	T	B	N	DC	P	NS (PS, ASD)	[49]
64	13	c.1530G > C	p.Q510H	112,926,910	rs397507550	24.5	D	PD	D	DC	P	LS (PS, ASD, HCM)	[54,55]
65	13	c.1510A > C	p.M504L	112,926,890	NA	23.1	T	B	N	DC	NA	NS	[43]

*CHD* congenital heart disease, *NS* Noonan syndrome, *LS* LEOPARD syndrome, *PS* pulmonary valve stenosis, *ASD* atrial septal defects, *VSD* ventricular septal defect, *AVSD* atrioventricular septal defect, *AVCD* atrioventricular canal defect, *PST* paroxysmal supraventricular tachycardia, *PDA* patent ductus arteriosus, *HCM* hypertrophic cardiomyopathy, *PVS* pulmonary vein stenosis, *HIS* hypertrophy interventricular septum, *DC* disease causing, *D* deleterious, *T* tolerated, *N* neutral, *B*, benign, *PD* probably damaging, *P* pathogenic, *B* benign, *NA* not available/not applicable

CADD, Phred ≤ 20: damaging; Phred > 20: natural

SIFT, score ≤ 0.05: deleterious; score > 0.05: tolerable

Polyphen-2, score = 0–0.15: benign; score = 0.15–0.85: possibly damaging; score = 0.85–1: probably damaging

PROVEAN, score ≤ − 2.5: deleterious; score > − 2.5: natural

#### Family I

The proband (III-3) was a 2-year-old girl diagnosed with CHD (Fig. 1A1). She was referred to Rajaie Cardiovascular Medical and Research Center to undergo WES. Computed tomography angiography was performed (Fig. 2A1). The proband of the first pedigree had severe cyanosis. A diagnostic workup showed that her 8-year-old brother (III-2) also had the same CHD. In both patients of this family, dysmorphology was evaluated by a medical geneticist, and no syndromic features were diagnosed. The inheritance pattern of the studied family was autosomal dominant (Fig. 1A1). Filtering the WES data yielded 2 variants as final candidate variants, probably responsible for CHDs in the family. In addition, PCR-based Sanger sequencing was performed on all available family members to confirm the identified variants observed in the family and to find a heterozygous stop-gain variant (NM_182925.5: c.C244T: p.R82X) in exon 3 of the *FLT4* gene in the studied proband and her affected brother. The heterozygous c.C244T variant in the *FLT4* gene was detected in her healthy mother (II-6), while her healthy father (II-5) lacked this variant (Fig.1 A2). The other nonsynonymous variant in the *TRRAP* gene did not segregate with the defects in the family. WES on the proband’s genomic DNA identified the variant in the *FLT4* gene as the genetic cause of CHDs in the family. According to the American College of Medical Genetics and Genomics 2015 (ACMG) [56], the c.C244T variant is a pathogenic variant (criteria: PVS1, PM2, PP1, PP4, and PP5). The stop-gain variant was considered the cause of the disease by MutationTaster and CADD. The sequence alignments of proteins showed that the stop-gain variant occurred within a highly conserved amino acid across various species, which supports its essential performance (Fig. 1A3).

#### Family II

The proband (III-2) was a 3-year-old girl diagnosed with CHD (Fig. 1B1). She was referred to Rajaie Cardiovascular Medical and Research Center to undergo WES. The computed tomography angiography results are displayed in Fig. 2B1. The proband of the second pedigree was cyanotic. The diagnostic workup showed that her 6-year-old sister (III-1) had the same CHD. In both patients of this family, dysmorphology was evaluated by a medical geneticist, and no syndromic features were diagnosed. The inheritance pattern of the second family was autosomal dominant (Fig. 1B1). Six variants were identified as final candidate variants, probably responsible for CHDs in the family. PCR-based Sanger sequencing confirmed the presence of the nonsynonymous variant (NM_002834: c.C1403T:p.T468M) in exon 12 of the *PTPN11* gene in the studied proband and her affected sister (Fig. 1B2). The affected proband (III-2) and her affected sister (III-1) were heterozygous for this locus, whereas her unaffected father (II-4) was heterozygous for this locus, and his unaffected mother (II-5) lacked the variant (Fig. 1B2). The other variants in 5 genes (*AKAP12*, *DNAH11*, *HNRNPC*, *NPHP4*, and *FBN1*) did not segregate with the defects in this family. WES on the proband’s genomic DNA detected a heterozygous nonsynonymous variant (c.C1403T:p.T468M) in the *PTPN11* gene, which co-segregated with the defect within the family. According to the ACMG 2015 [56], the c.C1403T variant is a pathogenic variant (criteria: PS3, PM1, PM2, PM5, PP1, PP2, PP3, PP4, and PP5). The nonsynonymous variant was considered the cause of the disease by SIFT, PolyPhen, PROVEAN, MutationTaster, and CADD. The amino acid residue 468 of the human protein-tyrosine phosphatase, non-receptor type 11 is highly conserved among vertebrates (Fig.1B3).

### Protein structure modeling

Docking was performed between the normal/mutant VEGFR3 and VEGFC by obtaining the 3D structure of the normal/mutant VEGFR3 from the SWISS-MODEL server and downloading the 3D structure of VEGFC from PDB (ID: 2X1W, resolution: 2.7 Å). Further, the HADDOCK scores of the normal and mutant VEGFR3 with VEGFC were − 55.0 ± 2.5 and − 61.6 ± 18.5, respectively. The docking results indicated that the binding affinity of the mutant VEGFR3 to VEGFC was reduced. The binding site of the normal VEGFR3 was surrounded by 16 amino acids, which formed 21 hydrogen bonds with VEGFC. Nonetheless, in the Arg82Ter mutant VEGFR3, only 3 amino acids (Glu37, Ser38, and Ile41) formed 5 hydrogen bonds with VEGFC. This stop-gain variant reduced the hydrogen bond and the affinity with VEGFC (Figs. 3 and 4). Docking was performed between the normal/mutant SHP-2 and Gab-1 by downloading the 3D structures of the human SHP-2 (normal ID: 7jvn, resolution: 1.92 Å and mutant ID: 4ohl, resolution: 2.4 Å) and Gab-1 (ID: 4qsy, resolution: 2.1 Å) from PDB. The HADDOCK scores of the normal/mutant SHP-2 with Gab-1 were − 28.0 ± 7.5 and − 25.1 ± 4.1, respectively. The normal SHP-2 had 4 hydrogen bonds with Gab-1, and the T468M mutant SHP-2 had 10 hydrogen bonds. This variant led to more hydrogen bonds and hydrophobic interactions at the surface of SHP-2 with Gab-1 and a higher binding affinity. This increased affinity weakened the N-SH2–PTP inter-domain interaction in the T468M mutant SHP-2 and enhanced the binding of N-SH2 with Gab-1 (Figs. 5 and 6).

## Discussion

In the present study, WES unraveled a novel stop-gain variant, which affected the *FLT4* gene, as well as a known nonsynonymous c.C1403T:p.T468M variant in the *PTPN11* gene. VEGFs regulate angiogenesis and vasculogenesis by binding to the receptor tyrosine kinases VEGFR1, VEGFR2, and VEGFR3 [57]. The *FLT4* gene encodes VEGFR3, which plays a unique role in the survival, proliferation, and migration of lymphatic endothelial cells. In adults, VEGFR3 is predominantly expressed in lymphatic endothelial cells and is crucial to lymphatic vessel growth [58]. Furthermore, this receptor is expressed in vascular endothelial cells during embryonic development, which is crucial to the development of blood vessels [59]. This human gene has 31 exons, which encode a protein with an extracellular region consisting of 7 immunoglobulin-like domains, 1 transmembrane region, 2 intracellular tyrosine kinase domains, and 1 c-terminal tail [24]. The *NOTCH1* and *FLT4* genes are the genes most frequently implicated in the etiology of the tetralogy of Fallot, with their variants accounting for almost 7% of all cases [60]. In a prior study, loss-of-function variants in the *FLT4* gene were found in 2.3% of the recruited patients with the tetralogy of Fallot [27].

A cohort study by Page et al. showed that the variants of the *FLT4* gene contributed to the incidence of the tetralogy of Fallot, accounting for 2.4% of the studied patients. In that study, 22 *FLT4* variants were reported in 21 patients with the tetralogy of Fallot. In addition, 16 of the variants identified were loss of function: 4 splice variants (c.3002-1C > T, c.3002-2 T > C, c.2300C > G, and c.2849del21), 6 indels resulting in frameshifts and premature truncation (p.P363fsX25, p.Q423fsX3, p.L636fsX3, p.Y853fsX20, p.N905fsX20, and p.Y1337fsX19), and 6 premature termination codons (p.Y361X, p.Y369X, p.E896X, p.Q920X, p.R1031X, and p.Q1126X) [59].

A genome-sequencing study conducted by Reuter et al. reported 9 novel variants in the *FLT4* gene related to the tetralogy of Fallot [19]. Elsewhere, a frameshift deletion in the *FLT4* gene in a patient with the tetralogy of Fallot was detected by WES [44]. A link has been reported between *FLT4* loss-of-function mutations and the tetralogy of Fallot, and VEGF signaling seems to be a new mechanism contributing to the pathogenesis of the disease [19, 59, 61]. The *FLT4* mutations identified in patients with the tetralogy of Fallot are mainly missense or truncating variants in extracellular domains. Nevertheless, all the *FLT4* mutations (missense or small in-frame deletions) known to cause Milroy disease have been located in 2 intracellular kinase domains (exons 17–26) and are assumed to interfere with the tyrosine kinase activation of the VEGFR3 receptor [15, 58]. The mutations within the tyrosine kinase domains of the VEGFR3 receptor lead to decreased tyrosine kinase activity [28]. The mutant receptor is maintained longer on the cell surface than is the wild type; thus, the amount of the mutant receptor on the surface of the endothelial cell is higher, which probably leads to lymphedema by reducing the relative rate of ligand binding to the active wild type [58]. Page et al. maintained that CHDs went unreported in patients with VEGFR3 variants causing Milroy disease [59]. In the current investigation, we conducted WES on a patient with CHD and succeeded in identifying a heterozygous stop-gain variant in the *FLT4* gene. Further, the pathogenic stop-gain variant (c.C244T: p.R82X) in the *FLT4* gene altered an arginine amino acid to a premature termination codon at codon 82, leading to the truncation of the VEGFR3 protein. The identified variant was not reported in the Genome Aggregation Database (gnomAD), the 1000 Genomes Project, ExAC, and ESP6500. This variant has been categorized in the Catalogue of Somatic Mutations in Cancer (COSMIC) as COSM126706. The C/T transition at position 244 in exon 3 predicted Arg82Ter within the VEGFR3 protein. The mutated residue was located in the VEGFR3 protein and disturbed its function by abnormally shortening it, with the amino acids lost affecting its main activity. Moreover, Arg82 is highly conserved among species, suggesting that the mutated amino acid is essential for protein function (Fig. 1A3). The detected variant in the *FLT4* gene can change the arginine amino acid to a premature termination codon at position 82 in the VEGFR3 protein, denoting a feasible mechanism for the pathologies associated with pulmonary atresia and ventricular septal defects. Most *FLT4* variants are associated with the tetralogy of Fallot; consequently, this CHD presents with a combination of defects such as ventricular septal defects, pulmonary valve stenosis, right ventricular hypertrophy, and the overriding aorta. In the present study, both patients of Family I had the identified *FLT4* variant, which is associated with large ventricular septal defects and pulmonary atresia with hypoplastic pulmonary artery branches and multiple aortopulmonary collaterals. This finding may indicate the diversity of the *FLT4* phenotype.

The *PTPN11* gene encodes SHP-2, which participates in signaling cascades downstream for cytokines and growth factors [62]. The mutations of the *PTPN11* gene have been identified in Noonan syndrome and LEOPARD syndrome [63]. In addition, the *PTPN11* gene is a candidate gene for contributions to the risk of the nonsyndromic tetralogy of Fallot [64]. Pulmonary valve stenosis is the most common CHD in Noonan syndrome patients with *PTPN11* mutations [40]. The *PTPN11* gene consists of 16 exons, which translate into a 593-amino acid protein with 4 distinct domains: 2 tandem SH2 domains (N-SH2 and C-SH2) on the N-terminal side, 1 protein-tyrosine phosphatase (PTP) domain, and 1 C-terminal hydrophilic tail on the C-terminal side [65]. The PTP domain and the 2 SH2 domains are the functional domains of the SHP-2 protein, and PTP activity is regulated by the SH2 domains (particularly N-SH2) [65, 66]. The N-SH2 domain works as a molecular switch in the SHP-2 protein, which interacts with the PTP domain in the inactive conformation, blocking the catalytic site [67]. *PTPN11* missense mutations were demonstrated to be gain-of-function changes that disrupted the intramolecular interaction between the PTP and N-SH2 domains, leading to increased SHP-2 activity [66]. The *PTPN11* mutations related to the N-SH2 and PTP domains can stabilize the active conformation of the SHP-2 protein [62]. The reported mutations in the *PTPN11* gene are clustered in 7 exons (2, 3, 4, 7, 8, 12, and 13) [68]. According to a study by Athota et al., the highest numbers of pathogenic variants were detected in exons 3, 8, and 13 [65]. The *PTPN11* mutations related to LEOPARD syndrome cluster in exons 7 and 12, and those related to Noonan syndrome cluster in exons 3 and 8 [69].

In the current study, using the WES facility, we presented an Iranian family with a known variant in the *PTPN11* gene. A medical geneticist confirmed nonsyndromic symptoms in the investigated family, who exhibited no features of Noonan syndrome. Furthermore, the c.C1403T variant in the *PTPN11* gene led to the substitution of methionine for threonine at position 468. The identified variant in the *PTPN11* gene was not reported in the 1000 Genomes Project and ESP6500. The variant has been cataloged in dbSNP as rs121918457 with a minor allele frequency of 0.000008236 in ExAC, with a minor allele frequency of 0.000004066 in gnomAD, and as COSM170715 in COSMIC. The C/T transition at position 1403 in exon 12 predicted the substitution of Met for Thr468 within the PTP domain of the SHP-2 protein. This residue is located in the protein tyrosine signature motif (positions 457–469), involved in phosphate binding. The mutated residue is located in the PTP domain, which plays a significant role in the main activity of the SHP-2 protein. The normal function of the SHP-2 protein can be consequently disturbed by the variant. Furthermore, the fact that Thr468 is highly conserved among species suggests that the mutated amino acid could be essential for protein function (Fig. 1B3). The detected variant in the *PTPN11* gene can change the amino acid at position 468 in the PTP domain, hinting at a feasible mechanism for the pathologies associated with pulmonary valve stenosis, ventricular septal defects, and atrial septal defects. In this study, the identified *PTPN11* variant was associated with nonsyndromic CHDs.

Concerning Family I, we examined the proband (III-3), her healthy parents, and her affected brother (III-2) with pulmonary atresia and a ventricular septal defect (Fig. 1A1). Regarding Family II, we examined the proband (III-2), her healthy parents, and her affected sister (III-1) with pulmonary stenosis, an atrial septal defect, and a ventricular septal defect (Fig. 1B1). In Family I, the 2 affected siblings had the same variant, while their mother was healthy despite having the same variant. In Family II, the 2 affected siblings had the same variant, while their father was healthy despite having the same variant. Phenotypic variability is observed in the members of such families with the same variants. Different phenotypes in individuals with the same variants can be caused by the incomplete penetrance of the phenotype. In both families, the asymptomatic parents of the affected children were unaffected carriers of the variants, indicating incomplete penetrance. Previous research suggests that the identified variants in the *FLT4* and *PTPN11* genes could cause the incomplete penetrance of the phenotypes [25, 59, 70]. Patients with the tetralogy of Fallot have inherited a variant of the *FLT4* gene from an asymptomatic parent, indicating that the mutant allele has reduced penetrance. Although the tetralogy of Fallot is seldom inherited in a Mendelian fashion, the penetrance of susceptibility variants is influenced by environmental and genetic factors [15, 59]. In the current study, we detected a novel pathogenic variant in the *FLT4* gene, which was associated with pulmonary atresia and ventricular septal defects in the first family. Identification of this novel variant via WES can improve the genetic diagnosis of cardiovascular diseases such as pulmonary atresia and ventricular septal defects. Notably, in the current investigation, we utilized molecular modeling to examine the molecular mechanism of the detected variant in the *PTPN11* gene for pathologies associated with pulmonary valve stenosis, ventricular septal defects, and atrial septal defects. Given the genetic heterogeneity of CHDs, finding the exact genetic cause is challenging. Despite a pattern of autosomal dominant inheritance in most families, genetic counseling in familial CHDs is complicated by reduced penetrance and variable expressivity [71]. Due to the hitherto unknown mechanisms involved in CHDs and the diversity in the nonsyndromic CHD spectrum, the molecular diagnosis of patients with such cardiac defects via WES could be deemed an appropriate, cost-effective approach [72, 73]. Additionally, our evaluation of these 2 families showed that WES was efficient in the accurate diagnosis of the genetic causes of CHDs with incomplete penetrance.

## Conclusions

The current study presents the first report of a novel pathogenic c.C244T variant in the *FLT4* gene, resulting in CHDs in an Iranian family. In addition, a heterozygous nonsynonymous c.C1403T variant in the *PTPN11* gene was identified through WES. Notably, WES is a preferable diagnostic implement for evaluating the complex genetics of CHDs.

## Data Availability

All data generated or analyzed during this study are included in this manuscript. The accession number of the identified variant in ClinVar is as follows: NM_182925.5 (FLT4):c.244C > T (p.Arg82Ter): VCV001177461.1.
